# Recent Advances in Pathophysiology and Therapeutic Approaches in Epilepsy

**DOI:** 10.3390/brainsci14080785

**Published:** 2024-08-02

**Authors:** Chandra Prakash, Pavan Kumar, Deepak Sharma

**Affiliations:** 1 Neurobiology Laboratory, School of Life Sciences, Jawaharlal Nehru University, New Delhi 110067, India; chandrabt87@gmail.com; 2 Department of Anatomy and Cell Biology, College of Medicine, The University of Illinois at Chicago, Chicago, IL 60607, USA; kumarpa@uic.edu

Epilepsy is a severe neurological disorder involving spontaneous and recurrent seizures, affecting a large number of people worldwide. According to estimates, about 150,000 patients are diagnosed with epilepsy every year, and at present, over 65 million people are affected by this debilitating neurological disorder [1]. The mechanism for the development and progression of epilepsy is diverse and is based on the epilepsy type and associated risk factors [2]. Current anti-seizure medications (ASMs) provide only symptomatic suppression of seizures, irrespective of pathophysiological alterations. Additionally, long-term usage of ASMs may result in adverse effects [3]. Thus, it is crucial to understand biochemical, structural, physiological, and molecular changes in epileptic brains. The search for novel therapeutic targets and treatment options is also warranted for diverse forms of epilepsy.

For this Special Issue, we invited authors with expertise in the pathophysiology and therapeutics of epilepsy to submit relevant, original research articles and review papers. We specifically sought articles addressing the progression of epilepsy in human patients and animal models, as well as those investigating new therapeutic approaches. As a result, twelve papers, including six research articles, five review articles, and one systematic review, were published in this Special Issue.

ASMs can cure the occurrence of seizures in children with epilepsy; however, some continue to have seizures and develop drug-resistant epilepsy (DRE) [4]. The retrospective study published by Liu et al. (contribution 1) found that resection surgery may be the most effective treatment option for DRE in children as it controls seizures and prevents intellectual disability. Nevertheless, patients for whom resection surgery was not an option could benefit from palliative surgery. The authors also suggested that children with DRE should receive treatment as soon as possible, regardless of the surgical treatment plan, to avoid developing brain damage and intellectual disability due to epilepsy.

Neuroinflammation is inherently accompanied by epilepsy, which can be targeted through disease-modifying medications [5]. Regarding this conjecture, Rabidas et al. (contribution 2) comprehensively reviewed the response of flavonoids to the neuroinflammatory process in different forms of experimental epilepsies. They concluded that flavonoids can reduce pro-inflammatory cytokines by modulating mediators like NF-kB and NLRP3 inflammasomes and other signaling molecules. Moreover, flavonoids can increase anti-inflammatory cytokines and decrease inflammatory mediators like COX-2, NOS, etc.; thus, they could be promising therapeutic agents for treating epilepsy.

Epilepsy occurring after a brain trauma is classified as post-traumatic epilepsy (PTE) and accounts for about 20% of total acquired epilepsy cases. Iron-induced epilepsy in rats is a widely used model for studying the pathophysiological mechanisms of PTE and its therapeutic interventions [6]. Research indicates that dehydroepiandrosterone (DHEA), an androgenic hormone, can be of therapeutic interest to epilepsy [7]. A research paper published by Prakash et al. (contribution 3) demonstrated that DHEA possesses antiepileptic and neuroprotective properties as it ameliorates astroglial activation, neuronal loss, and dendritic degeneration in the cortex and hippocampus regions of epileptic rats.

The mechanism of epilepsy appears to be connected with alterations in synapses, neurotransmitters, receptors, oxidative stress, mitochondria, cytokines, and apoptosis [8]. A review article by Madireddy and Madireddy (contribution 4) presented the availability of various therapeutic approaches, including ASMs and antioxidants, to treat epilepsy. Even though several ASMs are available, around one-third of patients with epilepsy continue to experience seizures. Individuals suffering from medically refractory epilepsy may experience reduced quality of life, cognitive impairments, and depressive symptoms. Other treatment options include surgery, neuromodulation, and dietary changes.

Patients with DRE with unilateral hemispheric pathology can benefit from surgical treatment, as previous reports show seizure freedom in patients with epilepsy [9]. The group of Del Gaudio et al. (contribution 5) published a case series reporting the safety and success of a modified vertical parasagittal hemispherotomy for hemispheric DRE. They suggested that this modified surgical procedure can help treat hemispheric DRE, as it has a high seizure freedom rate. Additionally, this procedure reduces postoperative hydrocephalus and improves patients’ motor and cognitive outcomes.

Status epilepticus (SE) is a medical emergency characterized by seizures that are prolonged or happen quickly, one after the other, with no recovery time [10]. Daytime-restricted feeding (DRF) is a period of intermittent fasting that exerts anticonvulsant properties through metabolic activation, epigenetic mechanisms [11], anti-inflammatory, and neuroprotective effects [12]. A research paper published by Mercado-Gómez et al. (contribution 6) reported that DRF reduces oxidative stress via Nrf2-mediated upregulation of antioxidant enzymes in a pilocarpine-induced acute seizure model. Furthermore, they demonstrated that DRE activates Nrf2 in astrocytes and can be used as a possible adjuvant treatment for SE.

When ASMs fail to control seizures on SE, they proceed to refractory SE, which further leads to super-refractory SE (srSE) if the seizures prolong longer than 24 h despite anesthesia [13]. A systematic review by Stavropoulos et al. (contribution 7) discussed how srSE in children may benefit from neuromodulation treatments such as deep brain stimulation, vagus nerve stimulation, and electroconvulsive therapy. Additionally, they recommended that, to avoid long-term neurologic complications, neuromodulation treatments should be considered at earlier stages of epilepsy.

The most common type of self-limited focal epilepsy is called self-limited focal epilepsy with centrotemporal spikes (SeLECTS). It is responsible for 6–7% of childhood epilepsy cases and affects children who had normal brain MRIs before seizure onset [14]. SeLECTS is an excellent model for examining the effects of interictal epileptic discharges (IEDs) on cognition due to its frequent and regular IEDs, low requirement for ASMs, and infrequent seizures [15]. A retrospective study that explored the relationship between IEDs and cognitive function was conducted by Dontaine et al. (contribution 8) on the SeLECTS cohort. The study showed that visuospatial skills for neuropsychological evaluations and qualitative EEG assessing the diffusion of focal spike waves to other brain regions should be included with standard quantitative EEG indices.

Transition readiness refers to the degree to which patients and members of their support system (parents, healthcare providers, etc.) can successfully transition from child-centered to adult-oriented healthcare [16]. Vacca et al. (contribution 9) proposed a comprehensive framework encompassing clinical and psychological aspects associated with the transition from childhood to adult medical care in patients with epilepsy. They concluded that increasing awareness of transition readiness is crucial to helping patients with epilepsy and their parents develop self-management skills. Anticipating the transition period may be useful in preventing problematic sleep patterns and fostering independence in health care management. Thus, the parents of patients with epilepsy and other rare disorders should be examined for their mental health, which can have an impact on their children’s well-being.

The development and progression of seizures involve multiple brain regions; thus, epilepsy can be considered a “network disease” [17,18]. A better understanding of epilepsy networks can help clinicians plan how to disrupt these networks and enhance surgical results. A review paper by Hines and Wu (contribution 10) demonstrated that exploring epilepsy networks enhances the understanding of pathophysiological developments and surgical outcomes. The removal of nodal networks implicated in epilepsy lowers the risk of seizure recurrence. The neuromodulation of various targets reduces seizure frequency while allowing exploration of important brain circuitry and white matter pathways. Finally, invasive diagnostics, including EEG, can provide critical information about epilepsy networks that helps guide future surgical decisions.

Another review article by Boleti et al. (contribution 11) discusses that though surgery is the most effective option for achieving long-term seizure freedom, it is only an option for patients who are unable to control their seizures with medication. Alongside medication intervention, non-pharmacological strategies can also be used; these include invasive and non-invasive neuromodulation therapies, a ketogenic diet, etc. Overall, a deeper understanding of the pathophysiology and etiology of epilepsy is necessary to identify new targets and clarify those already recognized, allowing for the development of newer treatments with fewer side effects and an even smaller influence on comorbidities.

Interictal spikes are inherently associated with behavioral and cognitive deficits in epilepsy as well as other psychiatric diseases [19]. However, most animal models exhibit both spikes and seizures, making it difficult to determine the precise role of interictal spikes in seizures and behavior. In this context, a contribution made by Eslami et al. (contribution 12) profoundly reviewed the investigations conducted on an interictal spiking model developed by tetanus toxin. Furthermore, they discussed that, although ASMs can decrease seizures, there is a scarcity of treatments targeting spike activity. The potential therapeutic targets can be identified for epileptic-spiking brain regions on the tetanus toxin model, which enables us to evaluate promising, novel treatments for clinical translation.

In summary, the articles collected in this Special Issue show the current research in the area of pathophysiological and therapeutic advancements in epilepsy. We believe that this Special Issue will significantly expand our current understanding of this field and help us develop novel treatment approaches for patients with epilepsy.
